# Isolated nodular hepatic tuberculosis in an immunocompetent patient following dexamethasone treatment for COVID-19

**DOI:** 10.1093/omcr/omaf030

**Published:** 2025-05-28

**Authors:** Jichang Seong, Egamberdiev Dilshod, Abdusattorov Ravshan

**Affiliations:** School of Medicine, Central Asian University, 264 Milliy, bog St, Tashkent 111221, Uzbekistan; Department of Hepatopancreatobiliary Surgery, Republican Specialized Scientific and Practical Medical Center of Oncology and Radiology, Farobi Street 5-7, Tashkent 100174, Uzbekistan; School of Medicine, Central Asian University, 264 Milliy, bog St, Tashkent 111221, Uzbekistan; Department of Oncology, AKFA Medline University Hospital, Tashkent city, Almazar district, st. Kichik Halka Yuli, 5A Tashkent 100211, Uzbekistan

**Keywords:** COVID-19, dexamethasone, extrapulmonary tuberculosis, granuloma, isolated nodular hepatic tuberculosis, tuberculoma

## Abstract

Hepatic tuberculosis is a rare extrapulmonary manifestation of pulmonary or miliary tuberculosis, typically seen in immunocompromised individuals. Isolated nodular hepatic tuberculosis is exceptionally uncommon in immunocompetent individuals and often mimics other hepatic lesions such as hepatocellular carcinoma. We present a 35-year-old male who developed isolated nodular hepatic tuberculosis following high-dose dexamethasone treatment for COVID-19. Imaging and fine-needle aspiration cytology suggested hepatocellular carcinoma, prompting surgical hepatic segmentectomy. Postoperative histopathological analysis confirmed the diagnosis of isolated nodular hepatic tuberculosis. This case highlights the potential risk of hepatic tuberculosis during temporary immunosuppression caused by high-dose dexamethasone treatment for COVID-19. Our case also emphasizes the importance of utilizing preoperative PCR and histopathological examination in diagnosing hepatic tuberculosis, helping to prevent unnecessary surgical interventions.

## Introduction

Hepatic tuberculosis (TB) often appears as an extrapulmonary manifestation of active pulmonary or miliary TB and is broadly categorized into two main types: the more common miliary (disseminated) form and the less common local (isolated) form, with further subclassifications into diffuse or nodular types [1, 2]. Immunocompromised patients, such as those with human immunodeficiency virus (HIV) or acquired immunodeficiency syndrome (AIDS), are particularly vulnerable to extrapulmonary TB and therefore, hepatic TB is exceptionally uncommon in an immunocompetent individual, making it challenging to distinguish from other hepatic lesions such as hepatocellular carcinoma and secondary metastases [1, 3, 4]. To date, only a number of cases of isolated hepatic TB in immunocompetent patients has been reported [5]. Emerging evidence suggests that COIVD-19 and its treatment, particularly immunosuppressive agents such as dexamethasone and prednisolone, may predispose patients to TB reactivation or new-onset TB, raising concerns about a potential TB resurgence in the post-COVID-19 era [6]. Here, we present a case of isolated nodular hepatic TB in an immunocompetent patient after COVID-19 treatment with dexamethasone and discuss its surgical management.

## Case presentation

A 35-year-old male presented to the hospital with complaints of chronic mild pain in the right upper quadrant, loss of appetite, and general malaise. His past medical history included treatment with high-dose dexamethasone for COVID-19 in 2020, after which his symptoms gradually began to appear over a couple of years. He denied having any chronic illnesses, such as diabetes mellitus. He had previously consulted a local doctor for symptom relief and was prescribed an herbal medicine, which he took for three months. During this period, he gained 15 kg but discontinued the herbal medicine due to leg edema and oliguria. He subsequently began losing weight again. Seeking the cause of his symptoms, he visited a local clinic where an abdominal ultrasound revealed focal hypoechogenic lesions in the liver measuring 27 × 22 mm and 27 × 16 mm. A follow-up examination at the same clinic after two months showed that the liver lesion has enlarged, now measuring 44 × 26 mm and 32 × 29 mm. With differential diagnoses including hemangioma, hepatocellular carcinoma, hydatid cyst, and amoebiasis, he was referred to our hospital for further investigation.

**Figure 1:**
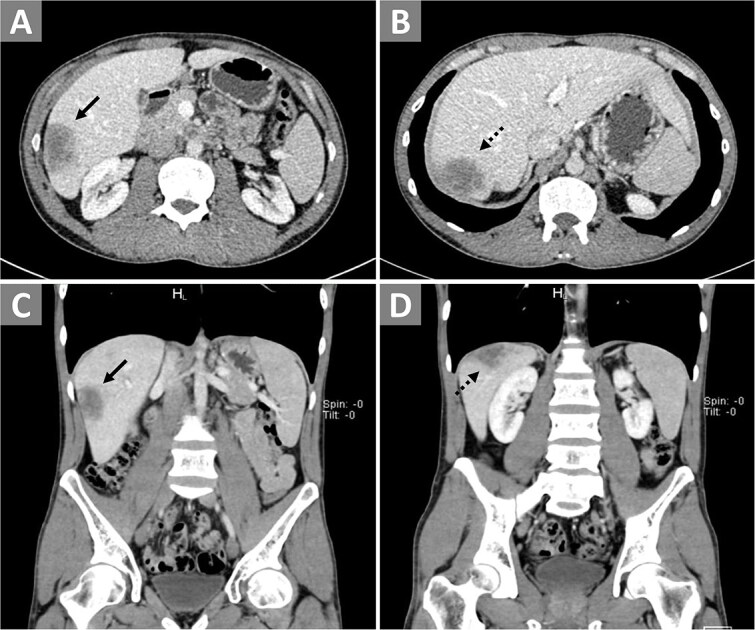
Contrast-enhanced CT of the abdomen. Panels (A) and (B) display transverse views, while panels (C) and (D) show coronal views. The solid arrows point to the lesion in the 6th segment, and dashed arrows indicate the lesion in the 7th segment of the right hepatic lobe.

On physical examination, his vital signs were within normal range with a body mass index (BMI) of 18 kg/m^2^. Liver and renal function tests and were within normal limits except for hypoalbuminemia. Tumor markers were within normal range, except for an elevated carcinoembryonic antigen (CEA). Enzyme-linked immunosorbent assay (ELISA) for parasitic infections, HIV, and hepatitis B/C were all negative (Table 1). Contrast-enhanced computed tomography (CT) of the chest and abdomen did not reveal any pathological findings except in the liver, where solid lesions with uneven margins were identified, measuring 30 × 44 × 40 mm in the 6^th^ segment (Fig. 1A and C) and 48 × 32 × 34 mm in the 7^th^ segment (Fig. 1B and D). Fine-needle aspiration cytology (FNAC) of the lesions showed dystrophic hepatocytes, raising suspicion of hepatic malignancy. Polymerase chain reaction (PCR) analysis for TB was not initially considered as the CT scan showed clear lungs with no pulmonary symptoms and the patient had no known history of TB. The imaging and FNAC findings were more indicative of hepatocellular carcinoma, leading to the decision for surgical management with segmentectomy.

**Table 1:** Blood and serum analysis results.

**Biochemical analysis**	**Unit**	**Result**	**Reference range**
Alanine aminotransferase (ALT)	U/L	35	0–40
Aspartate aminotransferase (AST)	U/L	15	0–40
Albumin	g/dL	33.2	35–55
Creatinine	μmol/L	102	44–115
Total bilirubin	μmol/L	12.2	1.7–20
Urea	mmol/L	3.2	2.9–8.2
**Tumor marker**	**Unit**	**Result**	**Reference range**
Alpha-fetoprotein (AFP)	ng/ml	3.2	<10
Cancer antigen (CA) 19-9	U/ml	30	<41
Carcinoembryonic antigen (CEA)	ng/ml	5.81	<5
**ELISA**	**Result**		
Amoebiasis	Negative		
Echinococcosis	Negative		
Toxoplasmosis	Negative		
Hepatitis B/C	Negative		
HIV	Negative		

**Figure 2:**
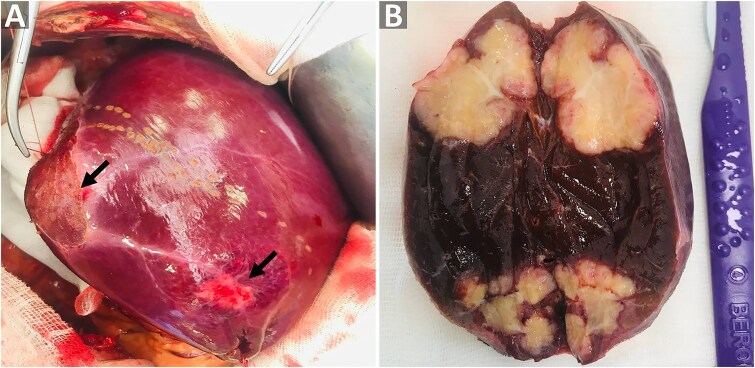
Intraoperative view of the lesions. (A) Gross view of the liver and lesions during surgery. (B) Cross sectional view of the resected liver showing the nodular tuberculomas. The black arrows point to the lesions located beneath Glisson’s capsule.

**Figure 3:**
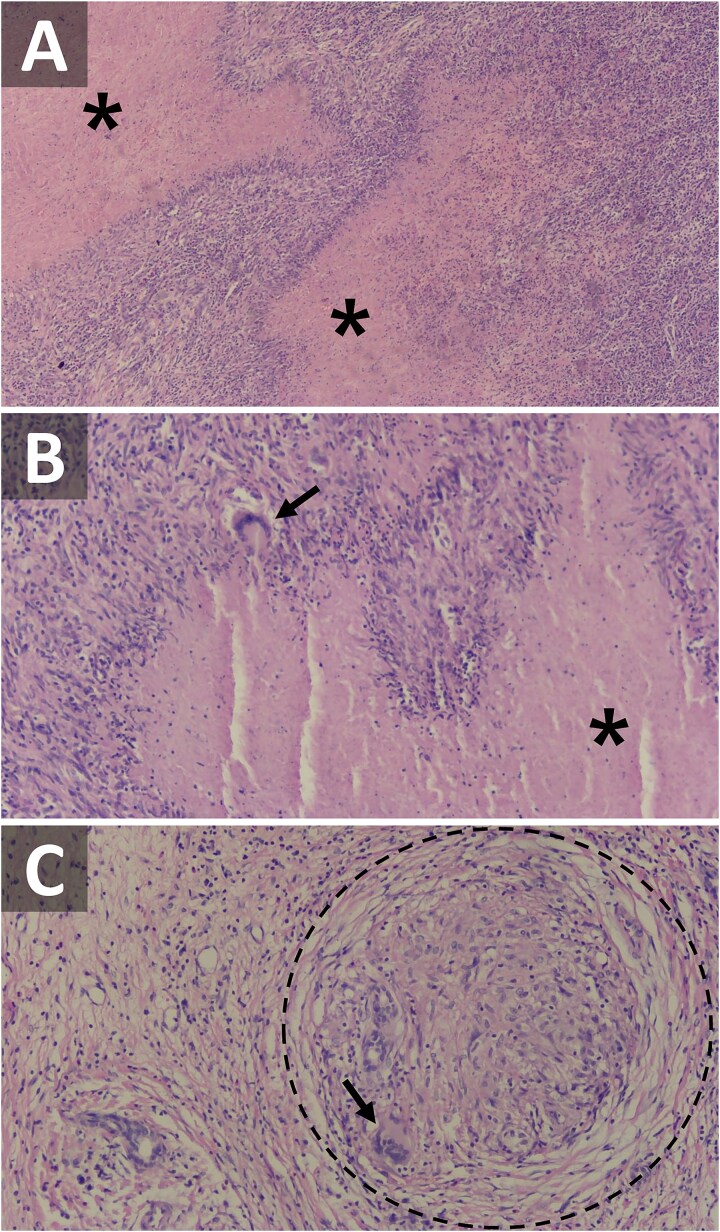
Hematoxylin and eosin (H/E) staining of the tuberculoma under a light microscope. (A) Low power (20x) and (B) and (C) high power (40x) magnifications. The black asterisks indicate areas of caseating necrosis, while the black arrows point to the Langhans giant cells. The dashed circle marks a granuloma.

During surgery, subcapsular lesions were observed in the right lobe of the liver without invasion of neighboring organs (Fig. 2A). Upon cutting the post-segmentectomy specimen, yellowish tumors with irregular borders were observed in the two segments of resected liver (Fig. 2B). Gross inspection of the lesions showed no signs of necrosis or purulent discharge. The surgery was completed successfully without significant complications. The specimen was then sent for histopathological analysis, which revealed lymphocytes and epithelioid cells surrounding central caseating necrosis (Fig. 3A and B), along with formation of Langhans giant cells and granulomas (Fig. 3C), confirming the diagnosis of TB. The patient was subsequently referred to an infectious disease specialist to initiate an anti-TB drug regimen.

## Discussion

Hepatic TB is extremely rare in immunocompetent individuals [3, 5] due to the low oxygen tension in the liver that provides unfavorable environment for the mycobacterial growth. Most cases of hepatic TB are reported in elderly or immunocompromised patients, particularly those with HIV infection or low socioeconomic status [2, 7]. Hepatic TB is broadly classified into miliary and local forms, which are further subclassified into diffuse and nodular types. It is believed that the miliary type results from the hematogenous spread of bacteria through the hepatic arteries, whereas the local type is likely seeded via the portal vein from the intestine [1, 2]. Notably, elevated CEA levels have been associated with intestinal TB, mimicking colorectal cancer [8]. Furthermore, a systematic review of 33 patients revealed that following COVID-19 infection, individuals were more susceptible to both reactivation of latent TB and development of new TB. This increased risk is likely attributed to the lung inflammation and temporary immunosuppression caused by COVID-19 infection itself, as well as the corticosteroid treatments used to manage it [6]. Although no intestinal pathologies were detected on our patient’s abdominal CT scan, we observed an elevated CEA level, which may indicate an intestinal route for TB. Therefore, we hypothesize that the patient developed hepatic TB following exposure to Mycobacterium-contaminated food during a period of temporary immunosuppression caused by high-dose dexamethasone treatment for COVID-19.

The timely and accurate diagnosis of hepatic TB remains challenging, as its manifestations are nonspecific [2, 4]. Common presenting symptoms of hepatic TB include right upper quadrant pain, fever, anorexia, weight loss, hepatomegaly, splenomegaly, and jaundice [9], while biochemical abnormalities often include elevated ALT and AST, and to a lesser extent, hypoalbuminemia and hyponatremia [2, 3]. In our case, neither the presenting symptoms nor the biochemical analyses were particularly informative, as the usual symptoms were largely absent, and most biochemical values fell within normal ranges.

While imaging studies such as ultrasonography (USG) and CT may assist in diagnosing hepatic TB, it is often indistinguishable from other hepatic lesions such as hepatocellular carcinoma and metastases based on imaging alone [4]. USG typically shows solitary or multiple hypoechogenic lesions without distinct wall, while CT scan shows non-enhancing, low-density lesions often with calcifications. Nevertheless, these features are also seen in hepatocellular carcinoma and metastatic lesions, making differentiation challenging [9]. Contrast-enhanced CT is considered the optimal imaging modality, while mycobacterial culture and biopsy remain the most specific diagnostic technique [2]. However, given the low rate of positive acid-fast staining and low sensitivity of mycobacterial culture, liver biopsy and histopathological examination is crucial for definite diagnosis of hepatic TB [2, 7, 9]. For example, Biswas et al. [10] reported that FNAC only yielded necrotic material and neither the acid-fast staining nor mycobacterial culture returned positive results. Conversely, histopathological examination revealed granulomas with caseating necrosis, confirming the diagnosis of hepatic TB [10]. In recent years, PCR has emerged as a more sensitive and specific diagnostic tool, providing faster results than mycobacterial cultures [2, 7]. Therefore, PCR may be performed preoperatively in suspected cases of hepatic TB to avoid invasive procedures [9].

Hepatic TB generally has a good prognosis and responds well to conventional anti-TB drugs, even without surgical interventions [9]. In TB-endemic areas, a course of anti-TB regimen may be initiated if granuloma is found on the liver biopsy [2]. However, surgical interventions such as segmentectomy or hepatectomy may be considered if the diagnosis is unclear, especially when the malignancy is suspected [9]. In our patient, the rapid growth of hepatic lesions and inconclusive FNAC results led to suspicion of hepatocellular carcinoma, necessitating surgical segmentectomy without performing PCR. Only postoperative histopathological analysis ultimately confirmed the diagnosis of isolated nodular hepatic TB.

## Conclusion

This report highlights that temporary immunosuppressive event, such as high-dose corticosteroid treatment for COVID-19, may predispose immunocompetent individuals to hepatic TB. In our case, inconclusive FNAC results shifted the diagnostic focus toward hepatocellular carcinoma, necessitating surgical segmentectomy. Preoperative PCR testing combined with histopathological examination may facilitate a definitive diagnosis, potentially preventing unnecessary surgical interventions due to misdiagnosis. Further research into the impact of COVID-19 and corticosteroid use on TB risk is crucial, especially in TB-endemic regions, to improve prevention strategies.

## Consent

Informed and written consent was obtained from the patient.

## Guarantor

Abdusattorov Ravshan.
